# High Circulating Platelet Count as a Risk Factor for Lung Squamous Cell Carcinoma: A Retrospective Study and Mendelian Randomization Analysis

**DOI:** 10.1111/crj.70090

**Published:** 2025-06-13

**Authors:** Guo‐Sheng Li, Jun Liu, Yue Li, Chang‐Qian Li, Dong‐Sheng Lu, Xiang Gao, Guan‐Qiang Yan, Zhan‐Yu Xu, Hua‐Fu Zhou, Nuo Yang

**Affiliations:** ^1^ Department of Cardiothoracic Surgery The First Affiliated Hospital of Guangxi Medical University Nanning Guangxi Zhuang Autonomous Region China

**Keywords:** biomarker, causality, lung cancer, Mendelian randomization

## Abstract

**Background:**

The association between platelet count (PLTC) and the risk of lung squamous cell carcinoma (LUSC) remains unclear.

**Methods:**

We analyzed 19 223 samples from public and internal cohorts to investigate the relationship between high PLTC and the risk of developing LUSC using the retrospective analysis and Mendelian randomization analysis (MRA).

**Results:**

Elevated PLTC were detected in the population with lung cancer compared to healthy individuals (odds ratio [OR] = 1.41 [per 1‐SD], 95% CI 1.13–1.75, *p* < 0.05). Furthermore, there is a significant association between elevated PLTC and an increased risk of LUSC based on an in‐house cohort (OR = 1.63 [per 1‐SD], 95% CI 1.08–2.45, *p* < 0.05). Individuals with high PLTC had an increased risk of developing LUSC using the inverse‐variance weighting method (OR = 1.62 [per 1‐SD], 95% CI 1.14–2.31, *p* < 0.05), an outcome that was directionally consistent across the weighted median, MR Egger, simple mode, and weighted modes methods (OR > 1.00). No pleiotropy (the MRA pleiotropy residual sum and outlier test *p* = 0.553) or heterogeneity (Cochran's *Q* statistic *p* = 0.777) was found in the MRAs. Besides PLTC, age and five other hematological parameters (e.g., red blood cell count) were identified as independent factors associated with the incidence of lung cancer or its subtype LUSC (*p* < 0.05).

**Conclusions:**

High circulating PLTC may serve as a risk factor for lung squamous cell carcinoma.

## Graphical abstract

1. A significant association was observed between genetically elevated platelet count and an increased risk of lung cancer and lung squamous cell carcinoma, based on the large sample sizes from the NHANES and in‐house cohorts.
2. Furthermore, our results indicate a causal effect of high platelet count on the occurrence of lung squamous cell carcinoma, which, to the best of our knowledge, has not been previously reported.

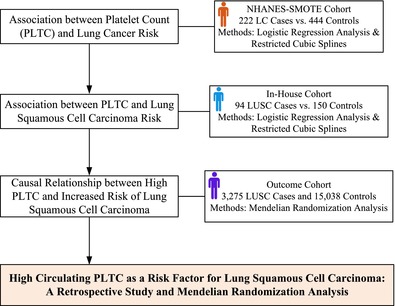

## 1 Introduction

Lung cancer (LC) is the leading cause of cancer‐related mortality worldwide [1]. Lung squamous cell carcinoma (LUSC) represents one of the most prevalent subtypes of LC and is frequently diagnosed at an advanced stage [2, 3, 4]. A substantial discrepancy exists in the 5‐year survival rates across different stages of LUSC: specifically, the 5‐year survival rates for patients with stage II and stage IV LUSC stand at 32% and 2%, respectively [4]. This disparity highlights the need to identify potential biomarkers for the early identification of individuals at elevated risk of developing LUSC.

While smoking remains the essential risk factor for LC [5], it is increasingly recognized that other factors, including genetic predisposition [6], play a significant role in the occurrence and development of the disease. Platelets (PLTs) play a crucial role in hemostasis, wound healing, and inflammation [7, 8]. Emerging evidence suggests that PLTs may also contribute to cancer progression and metastasis [9, 10]. Several studies have reported a positive association between platelet count (PLTC) and the risk of various malignancies, including LC [11]. However, the underlying cause of this relationship remains unclear.

Mendelian randomization analysis (MRA) leverages genetic variants—randomly allocated at conception and unaffected by confounders—as instrumental variables (IVs) to provide robust evidence of causality between exposures and outcomes [12, 13]. Given the relationship between PLTC and LUSC, MRA can help determine whether PLTC is a genuine risk factor for LUSC.

This study uses data from public and in‐house cohorts to explore the role of high PLTC in the incidence of LUSC. Furthermore, using summary‐level data from large‐scale genome‐wide association studies (GWAS) on PLTC and LUSC, this study aims to elucidate the potential causal relationship between high PLTC and the risk of LUSC using various MRA methods. These findings may inform LUSC prevention and treatment strategies, ultimately improving patient outcomes.

## 2 Materials and Methods

### 2.1 Collection of Data From National Health and Nutrition Examination Survey and Application of Synthetic Minority Oversampling Technique

National Health and Nutrition Examination Survey (NHANES) is a program designed to evaluate the health and nutrition status of the population from the United States. This survey provides information among several aspects, including demographic data and laboratory testing results. To initially explore the role of PLTC in LC, an NHANES cohort was collected from the NHANES database. The inclusion criteria were as follows: (1) age not less than 18 years old, (2) individuals who took part in the survey between 1999 and 2020, and (3) patients who provided definitive information on LC diagnosis. The exclusion criteria were as follows: individuals who did not participate in routine blood tests, including those lacking PLT‐related parameters (PLTC, mean platelet volume [MPV]), red blood cell‐related parameters (red blood cell count [RBC], mean corpuscular hemoglobin concentration [MCHC]), and parameters for the primary white blood cell types (absolute lymphocyte count [ALC], absolute monocyte count [AMC], and absolute neutrophil count [ANC]). The NHANES cohort consisted of 111 patients with LC and 46 406 healthy controls (Supplementary Material S1).

The Synthetic Minority Oversampling Technique (SMOTE) is an oversampling method used to address data imbalance, commonly applied in classification tasks where there is a large disparity between the number of minority and majority class samples [14, 15, 16]. This algorithm generates new samples through interpolation while controlling the number of majority class samples to achieve a more balanced cohort [15, 16]. Given the over 400‐fold difference in sample size between LC cases and controls in the NHANES cohort (111 versus 46 406), the SMOTE algorithm of the DMwR package [14] was utilized in this study. Here, SMOTE was used to double the number of minority class samples and adjust the majority class sample size to twice that of the minority class (1:2 ratio). The number of nearest neighbors was set to the default value of 5. A new NHANES‐SMOTE cohort was constructed using the SMOTE algorithm, comprising 222 LC cases and 444 controls (Supplementary Material 1). The NHANES‐SMOTE cohort and the original NHANES cohort were used in subsequent analyses. Moreover, to mitigate potential bias introduced by SMOTE's randomized sampling procedure, five additional replicate sampling iterations were performed to validate the NHANES‐SMOTE cohort findings (Supplementary Material 2). The parameters applied in these replicates were identical to those used in the original NHANES‐SMOTE cohort.

### 2.2 Collection of Internal Data

This study was approved by the Medical Ethics Review Committee of the First Affiliated Hospital of Guangxi Medical University (2021[KY‐E‐301]). To identify the role of PLTC in LUSC, an internal cohort was collected from the First Affiliated Hospital of Guangxi Medical University (FAH‐GXMU). The inclusion criteria were as follows: (1) age not less than 18 years old; (2) individuals who were seen between April 2019 and August 2023; and (3) patients whose LUSC was diagnosed by pathology. Exclusion criteria were consistent with those presented above for the NHANES cohort. The control group consisted of individuals who voluntarily underwent health check‐ups and had been ruled out of any lung diseases through computed tomography scans. Ultimately, the internal cohort included 94 patients with LUSC and 150 healthy individuals (Supplementary Material 3). The age, gender, as well as values of PLTC, MPV, RBC, MCHC, ALC, AMC, and ANC of the internal cohort were used to analyze the relationship between these clinical parameters and LUSC status.

### 2.3 Acquisition of Exposed and Outcome Data

Open GWAS data were obtained from the IEU Open GWAS project (https://gwas.mrcieu.ac.uk/). The exposure cohort (“ebi‐a‐GCST90002356”) [17] utilized in this study consisted of 15 171 samples and 34 208 859 single‐nucleotide polymorphisms (SNPs) associated with PLTC. The outcome cohort (“ieu‐a‐967”) [18] comprised 3275 LUSC cases and 15 038 control samples, encompassing 8 893 750 SNPs. Both the exposure and outcome cohorts represent individuals of European descent, and the data were employed for MRAs in this study.

### 2.4 Selection of IVs

We identified SNPs that were strongly associated with the exposure factor. IVs that were significantly associated with PLTC were selected through correlation analysis, with a filtering threshold of *p* value < 5 × 10^−8^. Those that exhibited a strong correlation with the exposure factor or explained a portion of its variance were selected using a filtering criterion of an *F*‐statistic > 10.

We removed linkage disequilibrium, that is, the tendency of genetic variants that are close in genomic position to be inherited together. This phenomenon increases the probability of alleles from different or multiple loci appearing together on a chromosome more than expected by chance. To ensure that the SNPs used for MRA were in complete linkage equilibrium, SNPs that were randomly distributed were selected by limiting the clumping distance within the region of linkage disequilibrium (*R* ^2^ < 0.001 within a 10 000‐kb clumping distance). We also identified the independence and exclusivity of SNPs. The relationship between IVs and phenotypes was explored using the Phenoscanner database (http://www.phenoscanner.medschl.cam.ac.uk/), and IVs associated with confounding factors causing LUSC were removed using the threshold of *p* value < 1 × 10^−5^. This ensured that the remaining IVs contributed only to the occurrence of LUSC through their effect on PLTC. Exclusivity was required for the IVs to act through the intermediate phenotype (i.e., PLTC) rather than directly causing LUSC, and the filtering criterion for retained SNPs in this process was a *p* value > 5 × 10^−5^.

### 2.5 MRAs Used in the Current Study

The estimated effect sizes of SNPs on exposure and outcome were calculated using inverse‐variance weighting (IVW), MR Egger, weighted median, simple mode, and weighted mode methods. IVW was considered the primary method and was of decisive value in determining the significance of the results. When IVW was found to be statistically significant and the trends of the other three methods were consistent with IVW, PLTC was identified as a factor influencing LUSC. We reported odds ratios (ORs) and 95% confidence intervals (CIs) to quantify the causal effect. An OR greater than 1 indicated that the exposure factor (high PLTC) was a detrimental factor for LUSC, while an OR less than 1 indicated that the exposure factor was a beneficial factor for the outcome.

### 2.6 Other Statistical Analysis

Logistic regression analysis was conducted to assess the associations between clinical parameters and the risk of LUSC. In this multivariate regression analysis, the presence of collinear variables can lead to unstable coefficient estimates. Stepwise regression, by automating the variable selection process, effectively identifies the variables with the strongest influence on the dependent outcome. Both multivariable logistic regression and stepwise selection included the following covariates: age, gender, and the hematologic parameters PLTC, MPV, RBC, MCHC, ALC, AMC, and ANC to evaluate their associations with LC status.

Restricted cubic splines (RCS) can flexibly capture and depict nonlinear relationships between independent and dependent variables, helping avoid the omission of potentially complex associations. Additionally, RCS allows for an intuitive display of a variable's dose–response relationship with the outcome through curve modeling. In this study, RCS was applied to explore the potential nonlinear relationship between PLTC and LUSC risk, as well as to examine their dose–response association.

**FIGURE 1:**
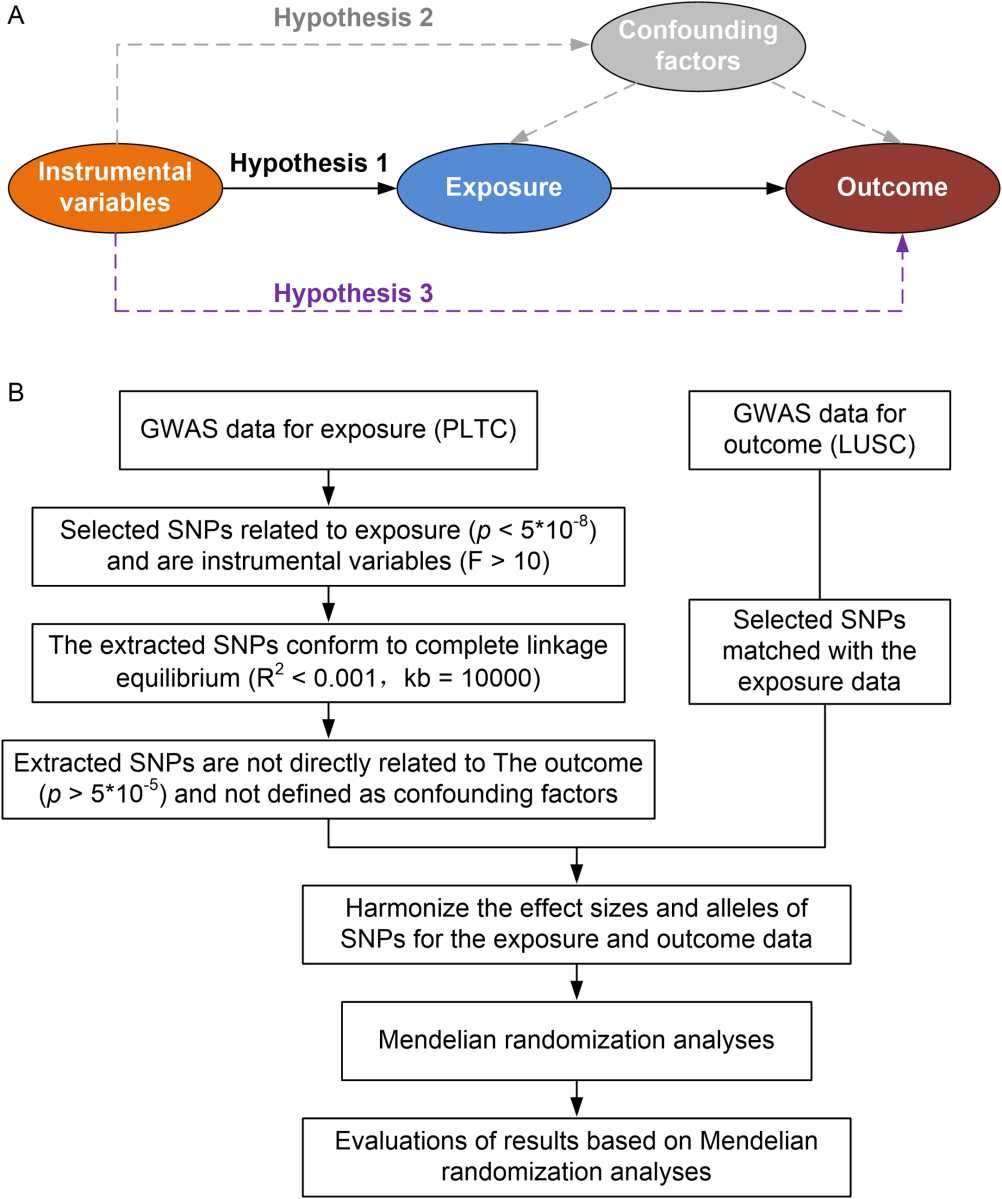
Overview of the Mendelian randomization analysis in this study. (A) Diagram illustrating the principles of the Mendelian randomization analysis. Instrumental variables should influence the exposure factor and subsequently affect the outcome (hypothesis 1), without direct effects through confounding factors (hypothesis 2). Furthermore, instrumental variables should not be directly associated with the outcome (hypothesis 3). (B) Flowchart of the Mendelian randomization analysis conducted in the study. PLTC, platelet count; LUSC, lung squamous cell carcinoma; SNP, single‐nucleotide polymorphism.

The level of pleiotropy in SNPs was assessed using the MRA pleiotropy residual sum and outlier test. If the *p* value was less than 0.05, the presence of pleiotropy in SNPs was suggested, indicating that PLTC may have simultaneously influenced the occurrence of LUSC through other factors. The heterogeneity of SNPs was tested using Cochran's *Q* statistic. A *p* value > 0.05 in the *Q* statistic test indicated absence of significant heterogeneity across SNP instruments. The leave‐one‐out method was used to detect whether each SNP had a driving effect on the MRA results, thereby assessing the robustness of the MRA results. Figure 1 presents a flowchart of the MRA pipeline. All calculations were performed using R (version 4.1.0) and several packages [14, 19, 20].

## 3 Results

### 3.1 The Relationship Between PLTC and LC by the NHANES Cohort

LUSC is one of the subtypes of LC; this study first investigated the association between PLTC and LC risk based on both original NHANES and NHANES‐SMOTE cohorts (Supplementary Material 1). In the original NHANES cohort, age and the levels of MCHC, ALC, AMC, and ANC were all significantly associated with LC risk (*p* < 0.05; Supplementary Material 4). However, since LC cases (*n* = 111) were outnumbered by controls (*n* = 46 406) by more than 400‐fold, this study instead employed the NHANES‐SMOTE cohort.

In the NHANES‐SMOTE cohort, adjusted ORs indicated that age and several hematologic factors (i.e., PLTC, MCHC, ALC, and AMC) were significantly associated with LC risk. Specifically, although no significant association was found for PLTC in the initial logistic regression analysis—likely due to collinearity issues, further stepwise regression analysis identified elevated PLTC as a risk factor for LC (per 1‐SD OR increase = 1.41, 95% CI 1.13–1.75, *p* = 0.002; Table 1). Similarly, as shown in Table 1, elevated AMC levels and older age were identified as risk factors for LC (per 1‐SD OR increase > 1.00, *p* < 0.05), while higher MCHC and ALC levels were identified as protective factors for LC (per 1‐SD OR increase < 1.00, *p* < 0.05).

**TABLE 1:** Associations of platelet count with lung cancer risk in the NHANES‐SMOTE cohort and lung squamous cell carcinoma risk in the in‐house cohort.

Cohort	Characteristics	Odd ratio (95% confidence interval, *p* value)	
		Logistic regression	Stepwise regression
NHANES‐SMOTE	Age	5.69 (4.12–7.86, *p* < 0.001)	5.77 (4.20–7.93, *p* < 0.001)
	Gender (male vs. female)	1.07 (0.68–1.68, *p* = 0.769)	
	PLTC	1.28 (0.99–1.65, *p* = 0.055)	1.41 (1.13–1.75, *p* = 0.002)
	MPV	0.86 (0.67–1.10, *p* = 0.224)	
	RBC	0.93 (0.74–1.18, *p* = 0.548)	
	MCHC	0.75 (0.60–0.95, *p* = 0.016)	0.79 (0.63–0.98, *p* = 0.032)
	ALC	0.81 (0.64–1.03, *p* = 0.080)	0.77 (0.61–0.96, *p* = 0.020)
	AMC	1.53 (1.01–2.31, *p* = 0.046)	1.73 (1.19–2.50, *p* = 0.004)
	ANC	1.17 (0.92–1.49, *p* = 0.210)	
FAH‐GXMU	Age	1.82 (1.18–2.80, *p* = 0.007)	1.78 (1.16–2.73, *p* = 0.008)
	Gender (male vs. female)	7.73 (3.28–18.24, *p* < 0.001)	
	PLTC	1.49 (0.96–2.31, *p* = 0.077)	1.63 (1.08–2.45, *p* = 0.020)
	MPV	1.68 (1.16–2.44, *p* = 0.006)	1.75 (1.22–2.51, *p* = 0.003)
	RBC	0.43 (0.26–0.72, *p* = 0.001)	0.45 (0.28–0.73, *p* = 0.001)
	MCHC	1.01 (0.67–1.52, *p* = 0.958)	
	ALC	0.40 (0.27–0.60, *p* < 0.001)	0.40 (0.27–0.59, *p* < 0.001)
	AMC	3.87 (1.50–10.01, *p* = 0.005)	4.87 (1.98–11.96, *p* < 0.001)
	ANC	1.39 (0.87–2.21, *p* = 0.165)	

*Note:* Stepwise regression analysis was conducted based on logistic regression analysis. NHANES‐SMOTE, National Health and Nutrition Examination Survey—Synthetic Minority Oversampling Technique. FAH‐GXMU, First Affiliated Hospital of Guangxi Medical University. PLTC, platelet count; MPV, mean platelet volume; RBC, red blood cell count; MCHC, mean corpuscular hemoglobin concentration; ALC, absolute lymphocyte count; AMC, absolute monocyte count; ANC, absolute neutrophil count.

**FIGURE 2:**
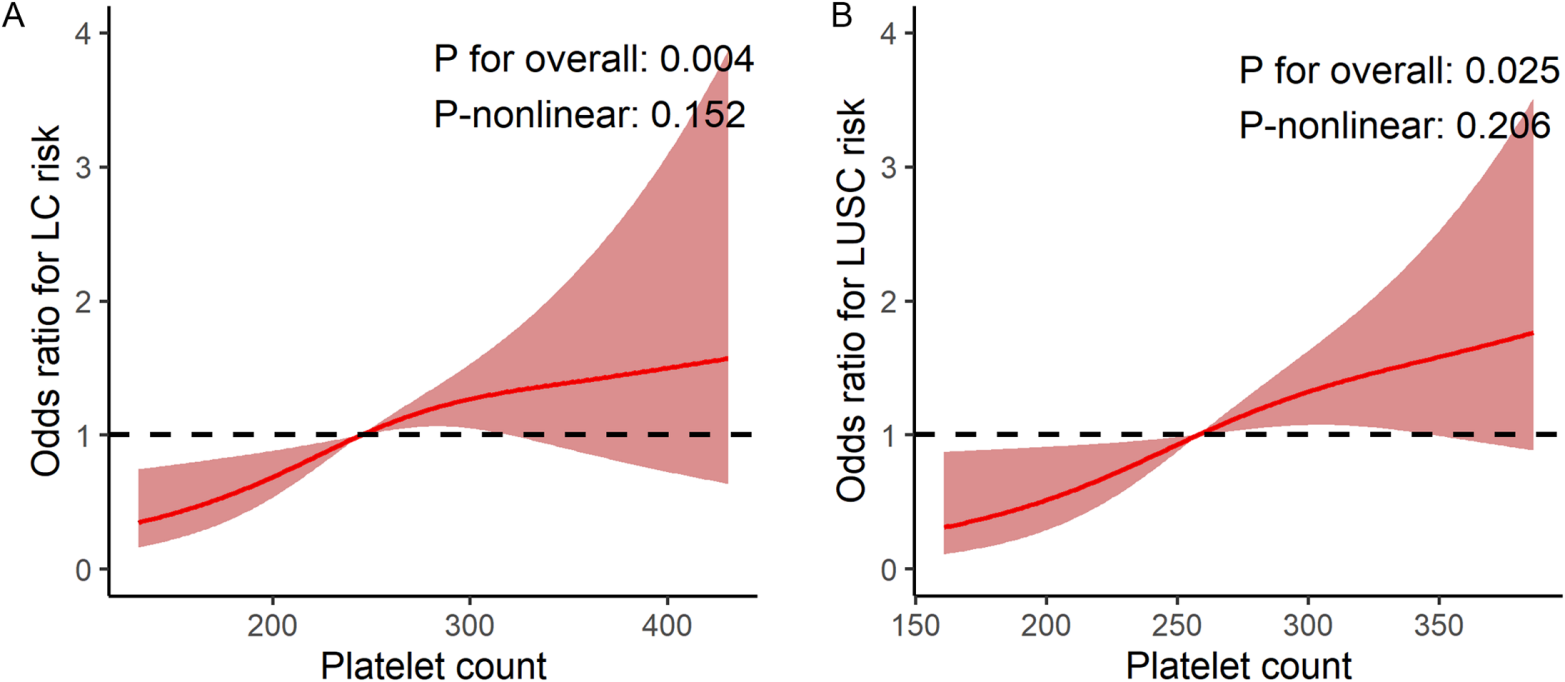
Restricted cubic splines illustrating the dose–response relationship between PLTC and lung cancer risk in the public cohort (A) and lung squamous cell carcinoma risk in the in‐house cohort (B). Splines were adjusted for age, gender, and other hematologic parameters (e.g., absolute lymphocyte count, absolute monocyte count) that achieved statistical significance in regression analyses. To better capture the dose–response relationship, odds ratio values are presented based on original data values rather than per 1‐standard deviation. The public cohort in this figure is National Health and Nutrition Examination Survey—Synthetic Minority Oversampling Technique cohort.

Furthermore, Figure 2A presents the RCS model illustrating the association between PLTC and LC risk, adjusted for MPV, RBC, MCHC, ALC, AMC, and ANC. This figure shows a significant dose–response relationship between PLTC and LC (*p* for overall = 0.004), suggesting a potential causal association. Additionally, no evidence of nonlinearity was detected (*p*‐nonlinear = 0.152, Figure 2A), which is consistent with the linear results obtained from the stepwise logistic regression analysis.

To assess robustness, both multivariable logistic regression and stepwise regression were performed across five independent NHANES‐SMOTE cohort replicates. Age and PLTC emerged consistently as risk factors for LC in all five replicates (per 1‐SD OR increase > 1.00, *p* < 0.05), whereas the significance of MCHC, ALC, and ANC was confirmed in at least two of the replicates (*p* < 0.05; Supplementary Material 5). Moreover, RCS analyses consistently indicated a linear association between PLTC and LC risk across all replicates (*p* for overall < 0.05, *p*‐nonlinear > 0.05; Supplementary Material 6).

### 3.2 The Association Between PLTC and LUSC by the FAH‐GXMU Cohort

We further determined the clinical role of PLTC in LSUC using the in‐house FAH‐GXMU cohort. The primary logistic regression analysis indicated that age and certain blood parameters (e.g., MPV, RBC, ALC, and AMC) were significantly associated with LUSC risk (*p* < 0.05; Table 1). Notably, the association between elevated PLTC and LUSC risk was confirmed by stepwise regression analysis (per 1‐SD OR increase = 1.63, 95% CI: 1.08–2.45, *p* = 0.020; Table 1). Additionally, age, MPV, and AMC levels remained significant risk factors (per 1‐SD OR increase > 1.00), while RBC and ALC were protective (per 1‐SD OR increase < 1.00) (*p* < 0.05; Table 1).

Using the RCS model, the overall association between PLTC and LUSC risk was statistically significant (*p* for overall = 0.025; Figure 2B), suggesting a positive correlation between PLTC and LUSC risk. Additionally, this relationship did not exhibit a significant nonlinear pattern (*p*‐nonlinear = 0.206; Figure 2B), implying a primarily linear association.

### 3.3 Selection of IVs for This Study

We further explored whether a potential causal correlation exists between them using the exposure and outcome cohorts (Supplementary Material 7). Thirteen IVs (rs75139539, rs9849502, rs1354034, rs34164109, rs210183, rs11772036, rs342299, rs10822159, rs11231642, rs78503944, rs138088102, rs8106212, and rs57843631) associated with PLTC were included in the exposure cohort. Among them, nine were strong IVs, with an *F*‐statistic of not less than 10. However, two IVs (rs210183 and rs10822159) were found to influence body mass index—a known causal factor for LUSC [21], and thus were excluded. The outcome cohort (ieu‐a‐967) included five IVs (Supplementary Material 8) for MRAs.

### 3.4 MRAs of PLTC on LUSC

**FIGURE 3:**
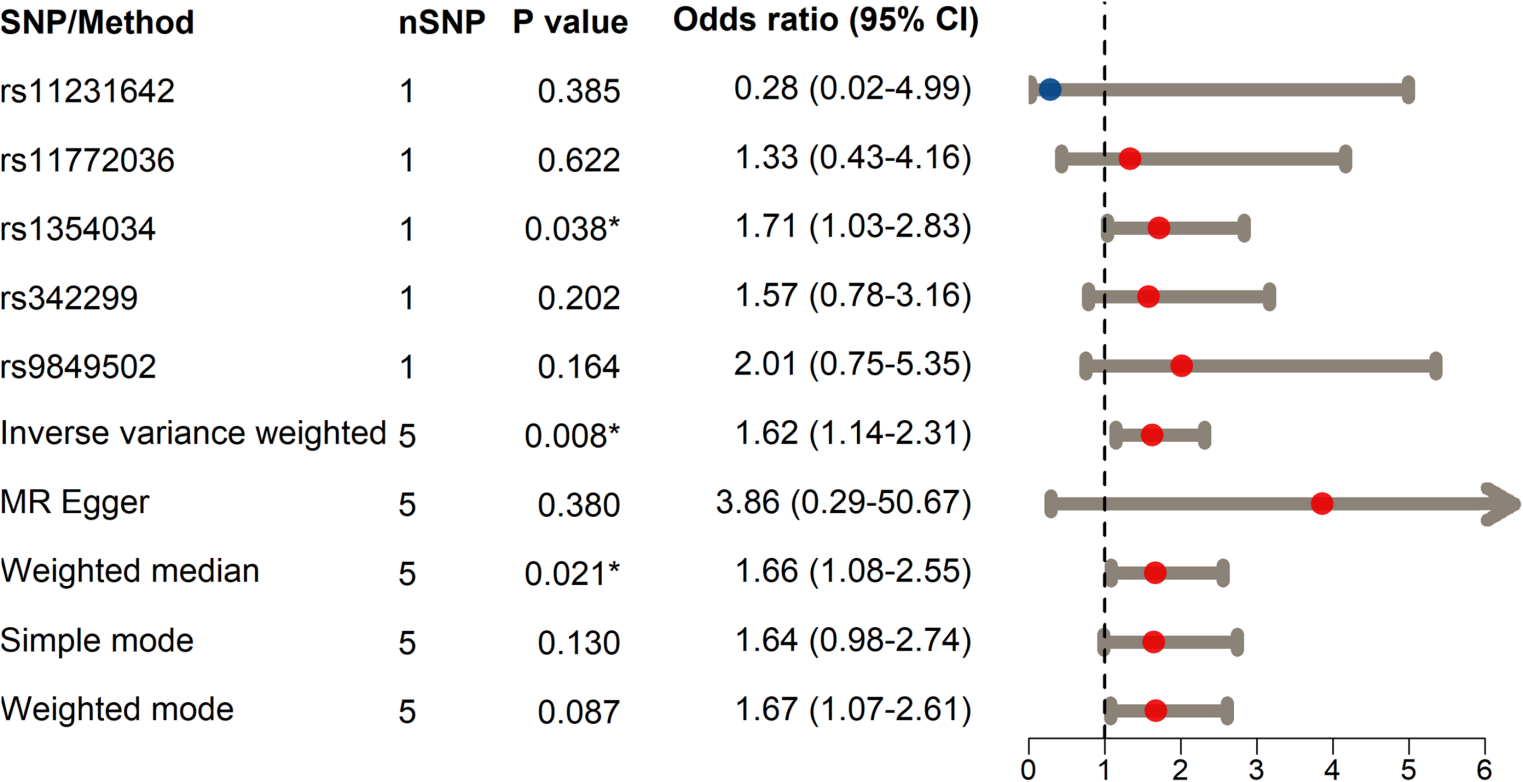
Evaluation of associations of higher PLTC with LUSC using single nucleotide polymorphism and Mendelian randomization analyses. CI, confidence interval. *p < 0.05.

Among the five SNPs (Table 2), rs1354034 was found to be independently associated with an increased risk of LUSC (β = 0.534, OR = 1.71, *p* = 0.038; Figure 3). The primary methods, IVW, of the MRA showed that individuals with high PLTC had an increased risk of developing LUSC (per 1‐SD OR increase = 1.62, 95% CI: 1.14–2.31, *p* = 0.008; Figure 3), an outcome that was directionally consistent across the weighted median (per 1‐SD OR increase = 1.66, 95% CI 1.08–2.55, *p* = 0.021; Figure 3). Although statistical significance was not reached in MR Egger, simple mode, and weighted modes, the directions of these methods were consistent with the results of the IVW and weighted median (per 1‐SD OR increase > 1.00, Figure 3), indicating that patients with high PLTC demonstrated a trend toward a greater risk of LUSC.

**TABLE 2:** Mendelian randomization analyses of platelet count on risk of lung squamous cell carcinoma (LUSC).

SNP/method	Exposure	Outcome	Sample size	Beta	SE	*P* value
rs11231642	PLTC	LUSC	18 313	−1.282	1.474	0.385
rs11772036	PLTC	LUSC	18 313	0.286	0.581	0.622
rs1354034	PLTC	LUSC	18 313	0.534	0.258	0.038*
rs342299	PLTC	LUSC	18 313	0.454	0.356	0.202
rs9849502	PLTC	LUSC	18 313	0.697	0.500	0.164
Inverse variance weighted	PLTC	LUSC	18 313	0.483	0.181	0.008*
MR Egger	PLTC	LUSC	18 313	1.350	1.314	0.380
Weighted median	PLTC	LUSC	18 313	0.507	0.219	0.021*
Simple mode	PLTC	LUSC	18 313	0.496	0.261	0.130
Weighted mode	PLTC	LUSC	18 313	0.514	0.228	0.087

Abbreviation: SE, standard error.

* *P* < 0.05.

### 3.5 Evaluation of MRA Results

**FIGURE 4:**
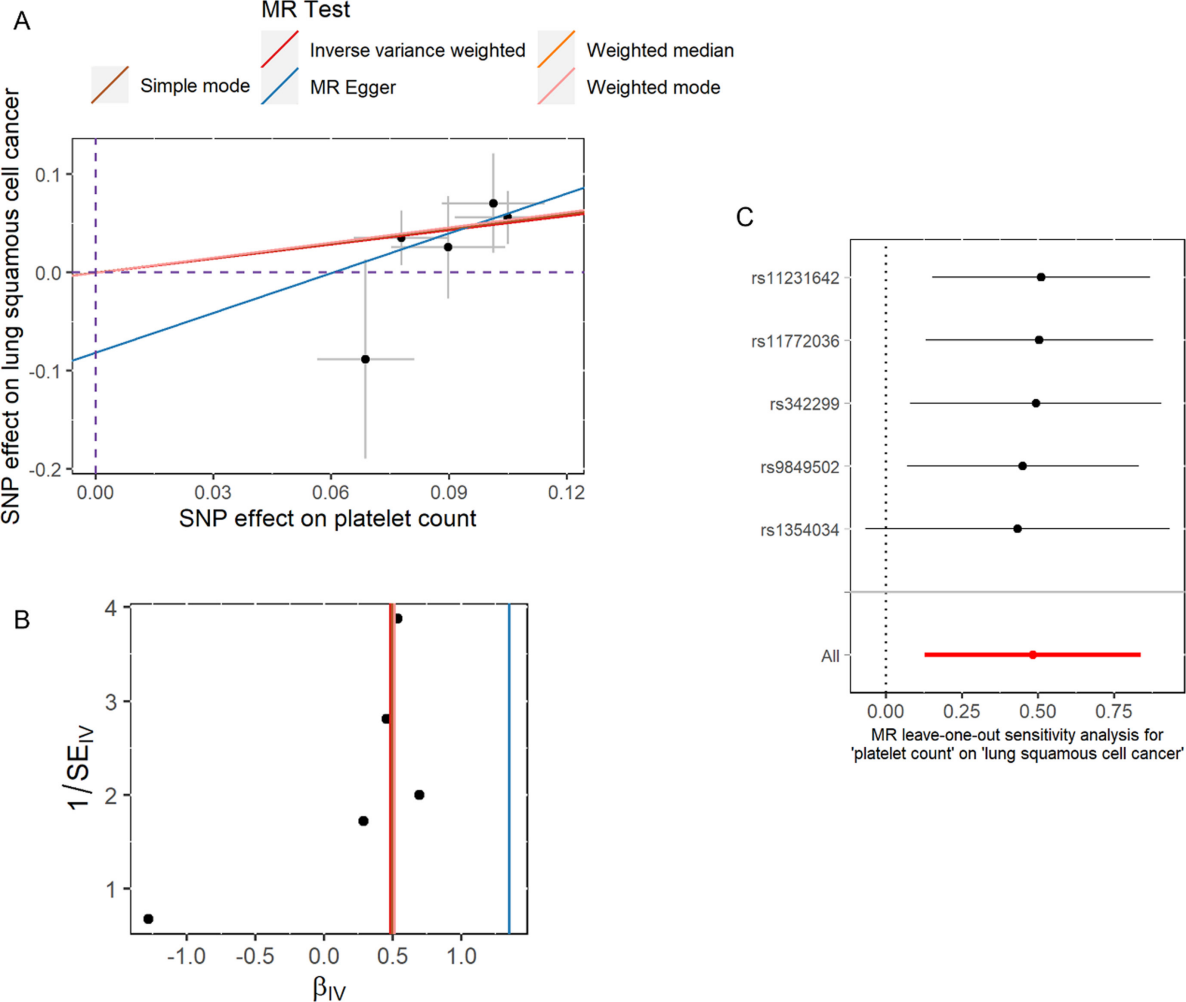
Evaluation of Mendelian randomization analysis results. (A) Scatter plot illustrating the effect of single nucleotide polymorphism on platelet count and LUSC. (B) Funnel plot for detecting heterogeneity. (C) Forest plot demonstrating the effect on lung squamous cell carcinoma driven by any single nucleotide polymorphism.

The scatter plot of SNP effects (Figure 4A) demonstrates a positive correlation between genetic associations for PLTC and LUSC risk, supporting a potential causal effect of PLTC on LUSC. The MRA did not reveal any signs of horizontal pleiotropy based on MRA pleiotropy residual sum and outlier analysis (*p* = 0.553). The scatter plot in Figure 4A further supports this observation, as the SNP effect estimates also cluster closely around the regression line through the origin. Furthermore, as depicted in Figure 4B, no heterogeneity was observed between the various SNPs from the results of the IVW analysis, which was supported by the heterogeneity statistics (Q value = 1.776, *p* = 0.777). Leave‐one‐out analysis indicated that no single SNP could negate the potential causal association between PLTC and LUSC (Figure 4C).

## 4 Discussion

In this study, we aimed to investigate the relationship between high PLTC and the risk of developing LUSC through retrospective analysis and MRAs. There is a significant association between genetically elevated PLTC and an increased risk of LC and LUSC based on the NHANES‐SMOTE and FAH‐GXMU cohorts. Furthermore, we utilized PLTC‐associated genetic variants as IVs to reduce confounding. Our results suggest the potential causal effect of high PLTC on LUSC occurrence, which, to the best of our knowledge, has not been previously reported. Moreover, age and certain hematological parameters are also identified to be correlated with the risk of LC and its subtype LUSC.

Elevated PLTC is increasingly recognized as a contributing factor in the onset of LC and as a predictor of its prognosis. Prior studies have indicated that individuals with higher PLTC levels are at significantly greater risk of developing LC compared to those with lower counts [22, 23], a relationship further substantiated by our findings. Notably, our logistic regression and RCS analyses suggest that this association follows a linear pattern. Consistent with these observations, multiple studies have shown a link between elevated PLTC and poorer prognosis in patients with LC [24, 25, 26], with models incorporating PLTC demonstrating robust predictive power for the prognosis of LC [27, 28]. Together, these findings underscore the potential role of PLTC as a key biomarker in the development and progression of LC, highlighting its utility for early detection and risk stratification.

High circulating PLTC represents a potential causal risk factor for LSUC. In a study conducted by Mounce et al. that analyzed a population aged 40 and over with normal PLTC levels, subjects with higher PLTC levels were found to be more likely to develop cancer than those with lower PLTC [29]. Similarly, a case–control study by Giannakeas et al. [23] indicated that higher PLTC levels were associated with an increased risk of developing various types of cancer, including colon, ovary, and stomach cancers. Zhu et al. [30] further revealed that high PLTC levels are a causative factor for small cell LC. However, they did not find an underlying causal relationship between PLTC and LUSC [30]. In contrast, our combined retrospective analysis and MRAs identified high PLTC as a potential causative factor for LUSC, providing insight into disease etiology.

PLTs may contribute to the progression of LUSC through multiple pathways. They play a crucial role in tumor growth and metastasis, with key mechanisms involving immunosuppression and tumor cell survival influenced by PLT α‐granule components, such as TGF‐β1 (transforming growth factor‐β1) and PLT factor 4 (PF4) [31]. TGF‐β1 is an immunosuppressive cytokine that, on the one hand, directly inhibits the anti‐tumor activity of innate immune cells, including dendritic cells and natural killer cells [32, 33]. On the other hand, TGF‐β1 promotes tumor development by suppressing key tumor cell cytokines, interleukin‐2, and interferon‐γ, which are critical for adaptive anti‐tumor immune responses [31]. Further, TGF‐β1 has been shown to inhibit the proliferation and differentiation of CD4+ and CD8+ lymphocytes, thereby facilitating cancer cell growth [11, 31, 34]. PF4, as a chemokine, induces megakaryopoiesis in a mouse model of lung adenocarcinoma. Specifically, overexpression of PF4 accelerates tumor initiation and progression, with PLTs playing a significant mediating role [35]. Overall, the current findings suggest a promotive role of PLTs in cancer. Given these characteristics of PLTs, it is reasonable to hypothesize that PLTC may be associated with these mechanisms as a causative factor for LUSC, although further experimental studies are needed to confirm this.

The public and internal cohorts included in this study have verified PLTC as a risk factor for LUSC, suggesting that targeting PLTC could represent a potential therapeutic strategy for treating LUSC. A viable therapeutic route may be through the signaling of interleukin‐6 (IL‐6), a cytokine that serves as a crucial driver for the hepatic synthesis of thrombopoietin; a decrease in IL‐6 expression can lead to a reduction in PLTC levels [36]. Interestingly, elevated levels of IL‐6 have been observed in several types of advanced LCs [37]. Therefore, targeting IL‐6 to reduce PLTC through drugs, such as tocilizumab, may represent one of the strategies for inhibiting LC. Targeting TGF‐β1, which is abundantly present in PLT α‐granules, may serve as another pathway to suppress the progression of LUSC by affecting PLTC. Recent clinical trials have demonstrated the efficacy of various promising TGF‐β1‐targeting drugs. For example, bintrafusp alfa is currently being tested as a first‐line treatment for patients with advanced non‐small cell LC (NSCLC, which includes LUSC) with high PD‐L1 expression [38]. Drugs that target TGF‐β1 have the potential to not only regulate the oncogenic activity of cells through PLTC but also reduce resistance to PD‐1/PD‐L1 immune therapy in patients with LC [39, 40], highlighting the significant value of these drugs in combating LC. Thus, focusing on PLTC may offer an effective approach for the prevention and treatment of LUSC.

In addition to PLTC, age and various hematological parameters may be linked to the risk of LC and its subtypes. Age has been identified as a prognostic risk factor for patients with NSCLC [41]. In patients with advanced NSCLC undergoing anti‐PD‐1 antibody therapy, elevated AMC is indicative of poorer prognosis [42]. Similarly, increased MPV adversely impacts LC prognosis [43]. Conversely, a higher MCHC correlates with a favorable LC prognosis [44]. Additionally, high ALC levels are associated with an absence of liver metastases in patients with NSCLC [45], suggesting that high ALC levels may impede the progression of NSCLC. These studies demonstrate the associations of five key indicators—age, AMC, MPV, MCHC, and ALC—with the clinical outcomes of LC or its subtypes. Our findings support the role of these parameters in influencing the risk of onset for LC or LUSC, validating previous research. Remarkably, our analysis also identified a previously unreported negative correlation between RBC and the risk of LUSC, underscoring the originality of this discovery.

There are several limitations to consider in light of the results of this study. First, our study focused only on LUSC, and the generalizability of our findings to populations with other types of cancers remains uncertain. Second, privacy concerns prevented us from precisely determining the timing of PLTC collection for NHANES samples obtained from public databases, so we could not collect PLTC data at diagnosis and prior to therapy for patients with LC. Prospective cohort studies will therefore be essential to establish a definitive causal relationship between PLTC and LUSC risk. Third, future experiments should also be performed to explore the mechanisms of PLTC in LUSC risk. Fourth, we did not collect prognostic information to explore a direct relationship between PLTC and the prognosis of patients with LUSC.

In conclusion, our study provides evidence supporting a potential causal relationship between high PLTC and an increased risk of LUSC. These findings highlight the importance of PLTC as a potential biomarker for LUSC risk stratification and prevention strategies.

## Author Contributions

GS.L., J.L., Y.L., ZY.X., HF.Z., and N.Y. conceived and designed the studies. GS.L., J.L., Y.L., CQ.L., DS.L., X.G., and GQ.Y. collected samples and performed calculations. GS.L., J.L., and Y.L. wrote the manuscript. All authors revised and approved the final manuscript.

## Ethics Statement

This study obtained approval from the Medical Ethics Review Committee of the First Affiliated Hospital of Guangxi Medical University (2021[KY‐E‐301]).

## Conflicts of Interest

The authors declare no conflicts of interest.

## Supporting information


**Supplementary Material 1** Characteristics of the National Health and Nutrition Examination Survey cohort in this study.
Supplementary Material 2 Characteristics of NHANES‐SMOTE cohort replicates.
Supplementary Material 3 Characteristics of the FAH‐GXMU cohort in this study.
Supplementary Material 4 Associations of PLTC with lung cancer risk in the original NHANES cohort.
Supplementary Material 5 Associations between PLTC and lung cancer risk in NHANES‐SMOTE cohort replicates 1–5. OR, odds ratio.
Supplementary Material 6 Restricted cubic splines illustrating the dose–response relationship between PLTC and lung cancer risk in NHANES‐SMOTE cohort replicates. Splines were adjusted for age, gender, and other hematologic parameters that achieved statistical significance in regression analyses. ORs are presented on the original measurement scale rather than per 1‐standard deviation to more accurately reflect the dose–response relationship.
Supplementary Material 7 Characteristics of the GWAS data included in this study.
Supplementary Material 8 Detailed information of platelet count‐specific single‐nucleotide polymorphisms (SNPs) selected for Mendelian randomization analysis.

## Data Availability

The data that support the findings of Mendelian randomization analysis are available in National Health and Nutrition Examination Survey (https://www.cdc.gov/nchs/nhanes/index.htm) and IEU Open GWAS project (https://gwas.mrcieu.ac.uk/) with serial number “ebi‐a‐GCST90002356” and “ieu‐a‐967.” Data on in‐house cohort are available from the corresponding authors upon reasonable request.
