# Supplementary material for: High Circulating Platelet Count as a Risk Factor for Lung Squamous Cell Carcinoma: A Retrospective Study and Mendelian Randomization Analysis
Source: Clin Respir J. 2025 Jun 13;19(6):e70090. doi: 10.1111/crj.70090 (PMC12164394; doi:10.1111/crj.70090)
Supplement: Supplementary file 1 — Supplementary Material 1 Characteristics of the National Health and Nutrition Examination Survey cohort in this study. Supplementary Material 2 Characteristics of NHANES‐SMOTE cohort replicates. Supplementary Material 3 Characteristics of the FAH‐GXMU cohort in this study. Supplementary Material 4 Associations of PLTC with lung cancer risk in the original NHANES cohort. Supplementary Material 5 Associations between PLTC and lung cancer risk in NHANES‐SMOTE cohort replicates 1–5. OR, odds ratio. Supplementary Material 6 Restricted cubic splines illustrating the dose–response relationship between PLTC and lung cancer risk in NHANES‐SMOTE cohort replicates. Splines were adjusted for age, gender, and other hematologic parameters that achieved statistical significance in regression analyses. ORs are presented on the original measurement scale rather than per 1‐standard deviation to more accurately reflect the dose–response relationship. Supplementary Material 7 Characteristics of the GWAS data included in this study. Supplementary Material 8 Detailed information of platelet count‐specific single‐nucleotide polymorphisms (SNPs) selected for Mendelian randomization analysis. [file CRJ-19-e70090-s001.pdf]

Supplementary Material 1. Characteristics of the National Health and Nutrition Examination Survey cohort in this study.

Clinical parameters	Original cohort		SMOTE cohort	
	Control (N=46406)	Lung cancer (N=111)	Control (N=444)	Lung cancer (N=222)
Gender				
Female	23990 (51.7%)	48 (43.2%)	226 (50.9%)	101 (45.5%)
Male	22416 (48.3%)	63 (56.8%)	218 (49.1%)	121 (54.5%)
Age (years)	48.12 ± 17.64	67.96 ± 11.69	46.08 ± 17.17	68.39 ± 10.78
PLTC (10^9/L)	252.84 ± 67.26	253.77 ± 75.47	254.19 ± 69.65	254.15 ± 67.25
MPV (fL)	8.21 ± 0.92	8.10 ± 1.06	8.24 ± 0.94	8.11 ± 1.00
RBC (10^{12}/L)	4.68 ± 0.51	4.53 ± 0.53	4.70 ± 0.54	4.53 ± 0.48
MCHC (g/dL)	33.83 ± 1.00	33.53 ± 0.88	33.85 ± 0.93	33.52 ± 0.85
ALC (10^9/L)	2.16 ± 0.94	1.81 ± 0.83	2.15 ± 0.67	1.83 ± 0.78
AMC (10^9/L)	0.56 ± 0.20	0.69 ± 0.62	0.55 ± 0.19	0.67 ± 0.50
ANC (10^9/L)	4.29 ± 1.80	4.94 ± 2.09	4.37 ± 1.77	4.86 ± 1.80

Notes: Data are presented as mean ± standard deviation. SMOTE, Synthetic Minority Oversampling Technique. PLTC, platelet count; MPV, mean platelet volume; RBC, red blood cell count; MCHC, mean corpuscular hemoglobin concentration; ALC, absolute lymphocyte count; AMC, absolute monocyte count; ANC, absolute neutrophil count.

Supplementary Material 2. Characteristics of NHANES-SMOTE cohort replicates.

Clinical parameters	Cohort replicate1		Cohort replicate2		Cohort replicate3		Cohort replicate4		Cohort replicate5	
	Control (N=444)	LC (N=222)	Control (N=444)	LC (N=222)	Control (N=444)	LC (N=222)	Control (N=444)	LC (N=222)	Control (N=444)	LC (N=222)
Gender										
Female	209 (47.1%)	98 (44.1%)	232 (52.3%)	104 (46.8%)	220 (49.5%)	99 (44.6%)	233 (52.5%)	102 (45.9%)	222 (50.0%)	105 (47.3%)
Male	235 (52.9%)	124 (55.9%)	212 (47.7%)	118 (53.2%)	224 (50.5%)	123 (55.4%)	211 (47.5%)	120 (54.1%)	222 (50.0%)	117 (52.7%)
Age (years)	47.60 ± 16.34	68.45 ± 10.44	47.20 ± 17.25	68.53 ± 10.76	47.98 ± 18.01	68.17 ± 11.29	48.73 ± 17.14	68.39 ± 10.77	47.81 ± 17.77	68.30 ± 10.63
PLTC (10^9/L)	251.59 ± 64.11	256.72 ± 69.93	250.06 ± 70.72	253.55 ± 69.04	250.99 ± 66.25	255.27 ± 70.39	251.62 ± 68.53	254.74 ± 69.43	252.12 ± 65.51	254.47 ± 67.44
MPV (fL)	8.15 ± 0.84	8.07 ± 0.95	8.20 ± 0.91	8.09 ± 0.99	8.20 ± 0.94	8.07 ± 0.94	8.18 ± 0.94	8.10 ± 0.94	8.22 ± 0.93	8.08 ± 0.93
RBC (10^{12}/L)	4.69 ± 0.49	4.53 ± 0.48	4.70 ± 0.49	4.51 ± 0.48	4.70 ± 0.51	4.52 ± 0.50	4.67 ± 0.52	4.52 ± 0.49	4.69 ± 0.51	4.53 ± 0.47
MCHC g/dL)	33.85 ± 1.00	33.52 ± 0.82	33.81 ± 0.93	33.50 ± 0.82	33.77 ± 1.04	33.53 ± 0.82	33.80 ± 1.02	33.50 ± 0.81	33.76 ± 0.89	33.49 ± 0.80
ALC (10^9/L)	2.18 ± 0.78	1.80 ± 0.76	2.15 ± 0.72	1.79 ± 0.79	2.15 ± 0.69	1.81 ± 0.77	2.15 ± 0.74	1.78 ± 0.76	2.14 ± 0.74	1.78 ± 0.77
AMC (10^9/L)	0.56 ± 0.19	0.68 ± 0.58	0.55 ± 0.18	0.67 ± 0.59	0.56 ± 0.20	0.67 ± 0.59	0.56 ± 0.21	0.66 ± 0.47	0.57 ± 0.20	0.68 ± 0.56
ANC (10^9/L)	4.24 ± 1.71	4.89 ± 1.83	4.17 ± 1.63	4.78 ± 1.74	4.26 ± 1.93	4.86 ± 1.92	4.25 ± 1.69	4.90 ± 1.86	4.29 ± 1.79	4.83 ± 1.74

Notes: Data are presented as mean ± standard deviation. LC, lung cancer.

Supplementary Material 3. Characteristics of the FAH-GXMU cohort in this study.

Clinical parameters	Control (N=150)	Lung squamous cell carcinoma (N=94)
Gender		
Female	75 (50.0%)	13 (13.8%)
Male	75 (50.0%)	81 (86.2%)
Age (years)	52.45 ± 14.59	61.59 ± 8.99
PLTC (10^9/L)	262.68 ± 67.09	272.66 ± 93.88
MPV (fL)	8.36 ± 0.88	8.64 ± 1.39
RBC (10^{12}/L)	4.69 ± 0.51	4.44 ± 0.70
MCHC (g/L)	330.68 ± 8.27	329.94 ± 12.67
ALC (10^9/L)	2.27 ± 0.63	1.83 ± 0.63
AMC (10^9/L)	0.48 ± 0.16	0.67 ± 0.56
ANC (10^9/L)	3.65 ± 1.16	4.42 ± 1.75

Notes: Data are presented as mean ± standard deviation. FAH-GXMU, First Affiliated Hospital of Guangxi Medical University.

Supplementary Material 4. Associations of PLTC with lung cancer risk in the original NHANES cohort.

Characteristics	Odd ratio (95% confidence interval, *p* value)	
	Logistic regression	Stepwise regression
Age	2.99 (2.33-3.84, *p*<.001)	3.05 (2.39-3.89, *p*<.001)
Gender (Male vs. female)	1.34 (0.89-2.03, *p*=.165)	
PLTC	1.11 (0.93-1.33, *p*=.247)	1.13 (0.96-1.32, *p*=.141)
MPV	0.91 (0.74-1.12, *p*=.371)	
RBC	0.92 (0.76-1.11, *p*=.365)	
MCHC	0.77 (0.64-0.94, *p*=.009)	0.80 (0.67-0.97, *p*=.023)
ALC	0.56 (0.42-0.76, *p*<.001)	0.53 (0.40-0.72, *p*<.001)
AMC	1.30 (1.15-1.48, *p*<.001)	1.32 (1.16-1.49, *p*<.001)
ANC	1.13 (1.03-1.24, *p*=.009)	1.13 (1.04-1.24, *p*=.006)

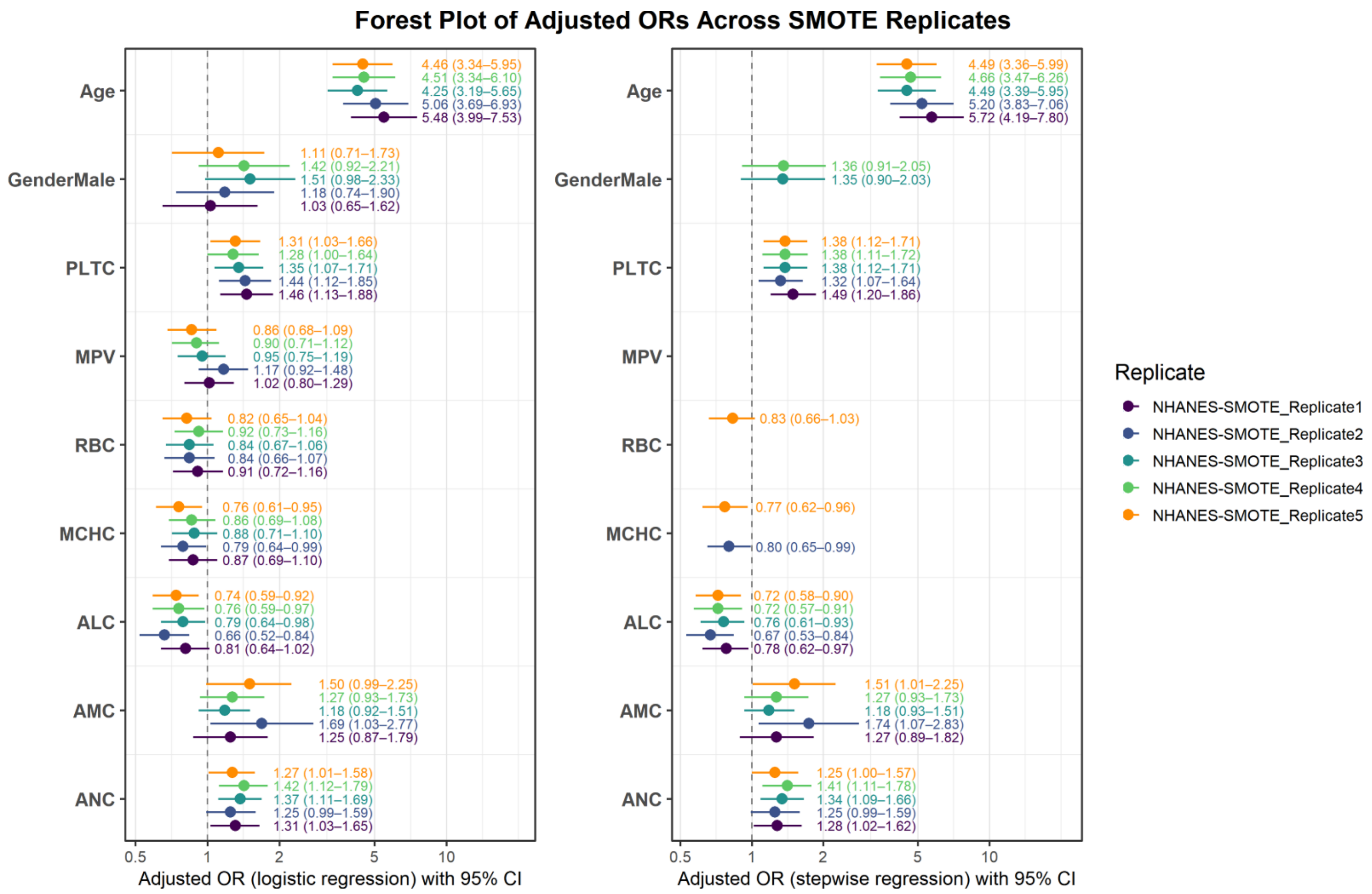

Supplementary Material 5. Associations between PLTC and lung cancer risk in NHANES-SMOTE cohort replicates 1–5. OR, odds ratio.

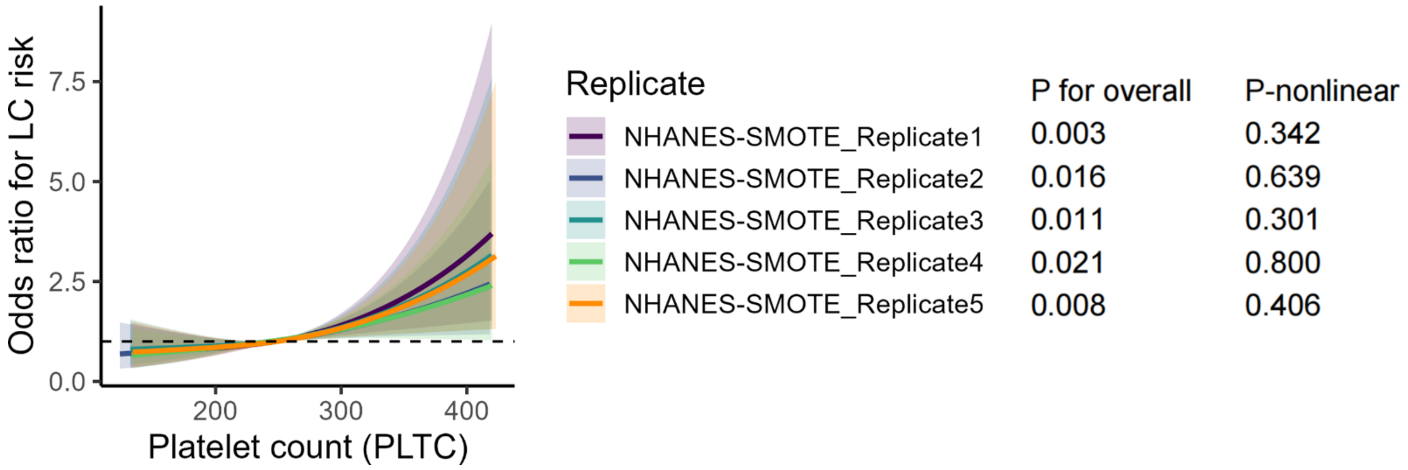

Supplementary Material 6. Restricted cubic splines illustrating the dose–response relationship between PLTC and lung cancer risk in NHANES-SMOTE cohort replicates. Splines were adjusted for age, gender, and other hematologic parameters that achieved statistical significance in regression analyses. ORs are presented on the original measurement scale rather than per 1-standard deviation to more accurately reflect the dose–response relationship.

Supplementary Material 7. Characteristics of the GWAS data included in this study.

GWAS ID	Sample size	Number of SNP	PMID
ebi-a-GCST90002356	15,171	34,208,859	32888493
ieu-a-967	3,275 cases vs. 15,038 controls	8,893,750	24880342

Supplementary Material 8. Detailed information of platelet count-specific single-nucleotide polymorphisms (SNPs) selected for Mendelian randomization analysis.

**Phenotype**	**Chromosome**	**SNP**	**Effect allele**	**Beta**	**Standard error**	***P***
PLTC	7	rs11772036	A	0.026	0.052	0.632
PLTC	7	rs342299	T	-0.035	0.028	0.232
PLTC	11	rs11231642	A	-0.088	0.101	0.390
PLTC	3	rs1354034	C	0.056	0.027	0.057
PLTC	3	rs9849502	T	0.071	0.051	0.154
